# Impact of Sexual Dimorphism on Trauma Patterns and Clinical Outcomes of Patients with a High-Risk Score of the Osteoporosis Self-Assessment Tool for Asians: A Propensity Score-Matched Analysis

**DOI:** 10.3390/ijerph15030418

**Published:** 2018-02-27

**Authors:** Chien-En Tang, Hang-Tsung Liu, Pao-Jen Kuo, Yi-Chun Chen, Shiun-Yuan Hsu, Chih-Che Lin, Ching-Hua Hsieh

**Affiliations:** 1 Department of Surgery, Kaohsiung Chang Gung Memorial Hospital and Chang Gung University College of Medicine, Kaohsiung 83301, Taiwan; chienen0707@gmail.com (C.-E.T.); immunologylin@gmail.com (C.-C.L.); 2 Department of Trauma Surgery, Kaohsiung Chang Gung Memorial Hospital and Chang Gung University College of Medicine, Kaohsiung 83301, Taiwan; htl1688@yahoo.com.tw; 3 Department of Plastic Surgery, Kaohsiung Chang Gung Memorial Hospital and Chang Gung University College of Medicine, Kaohsiung 83301, Taiwan; bow110470@gmail.com (P.-J.K.); libe320@yahoo.com.tw (Y.-C.C.); ah.lucy@hotmail.com (S.-Y.H.)

**Keywords:** trauma registry system, Osteoporosis Self-Assessment Tool for Asians (OSTA), male, female, osteoporosis, mortality, outcome

## Abstract

The Osteoporosis Self-assessment Tool for Asians (OSTA) is a validated index based on age and weight to predict the risk of osteoporosis in women. This cross-sectional study was designed to evaluate the impact of sexual dimorphism on the trauma patterns and the clinical outcomes of patients with high-risk OSTA scores. Trauma data of patients with high-risk OSTA scores between 1 January 2009 and 31 December 2015 were retrieved from the trauma registry system of a level I trauma center. A total of 2248 patients including 1585 women and 663 men were included in this study. In-hospital mortality was assessed as the primary outcome in the propensity score-matched analyses of the female and male patients, which were created in a 1:1 ratio under the adjustment of potential confounders, including age, co-morbidity, mechanism and injury-severity score (ISS). Female patients with a high-risk OSTA score had significantly lower mortality rates than their male counterparts. Among the propensity score-matched population, female patients had lower odds of having cerebral contusion and pneumothorax, but higher odds of presenting with radial, ulnar and femoral fractures than male patients. In addition, the female patients still had significantly lower odds of mortality (odds ratio (OR), 0.5; 95% confidence interval (CI), 0.29–0.90; *p* = 0.019) than the male patients. However, no significant differences were noted in the length of stay (LOS) in hospital, intensive-care unit (ICU) admission, and LOS in the ICU between the sexes. Female patients with high-risk OSTA scores showed different injury patterns and significantly lower mortality rates than their male counterparts, even after controlling for potential confounding factors.

## 1. Background

Osteoporosis is a disease characterized by loss of bone mass and density that has become more common with the rapidly increasing ageing population. With osteoporotic fractures being a significant factor in morbidity and mortality, osteoporosis has rapidly increased and become a widespread public health problem worldwide [1,2]. The gold standard for assessing bone density is dual-energy X-ray absorptiometry (DEXA). However, because of its relatively high cost, DEXA is not routinely used when screening for osteoporosis. Therefore, the World Health Organization (WHO) developed a simple screening tool, the Osteoporosis Self-assessment Tool for Asians (OSTA), in order to evaluate the risk of osteoporosis. The OSTA is an index that is based on age and weight and can be calculated using the following formula: (body weight (kg)—age (year)) × 0.2 [3]. Based on their risk of developing osteoporosis, patients are distributed into the following 3 categories: high-risk (OSTA score < −4), medium-risk (−1 ≥ OSTA score ≥ −4), and low-risk (OSTA score > −1). Patients categorized as high risk, medium risk, and low risk had 61%, 15%, and 3% risk of developing osteoporosis, respectively [3,4]. The OSTA score has been validated as an effective and feasible screening tool to identify patients at risk of developing osteoporosis in many Asian countries, including India, China, Korea, Japan, the Philippines, Malaysia and Taiwan [4,5,6,7,8,9,10,11].

Differences in trauma etiology as well as in physiological and behavioral characteristics between sexes had been reported [12,13]. Previous studies have shown that men when compared to women display an increased risk of presenting trauma injuries owing to their stronger inclination to engage in risky behaviors such as the consumption of alcohol or drugs, speeding, and violent action [14,15]. Thus, higher risk of injury-related mortality and morbidity are observed in men [16]. Reports from the United States have shown that men were at least 2.2 times more likely to sustain traumatic injury than women, with age, injury-severity score (ISS), and blunt-injury type being identified as the independent predictors of in-hospital mortality [17]. In addition, female motorcycle riders were found to have different injury characteristics and bodily injury patterns as well as lower ISS and in-hospital mortality than their male counterparts [12]. Currently, although many studies have used the OSTA score to investigate the clinical presentation and its associated outcome of patients with osteoporosis [9,18,19,20,21,22,23], few studies have analyzed the differences between men and women. Therefore, the aim of this study was to compare the differences in trauma patterns and clinical presentations between male and female trauma patients with high-risk OSTA scores at a level I trauma center. Furthermore, this study used a propensity score-matched analysis to assess and compare the outcomes between the sexes after eliminating confounders such as age, comorbidity, mechanism and ISS.

## 2. Methods

### 2.1. Ethical Considerations

After obtaining approval (approval number: 201600352B0 and 201600348B0) from the institutional review board (IRB) of Chang Gung Memorial Hospital, a level I trauma center located in southern Taiwan [12,24], we reviewed all patients enrolled in our trauma registry system between 1 January 2009 and 31 December 2015. The analyses were conducted using anonymized secondary data without linking the information to an individual patient. Informed consent was waived according to the regulations of the IRB.

### 2.2. Study Population

**Figure 1:**
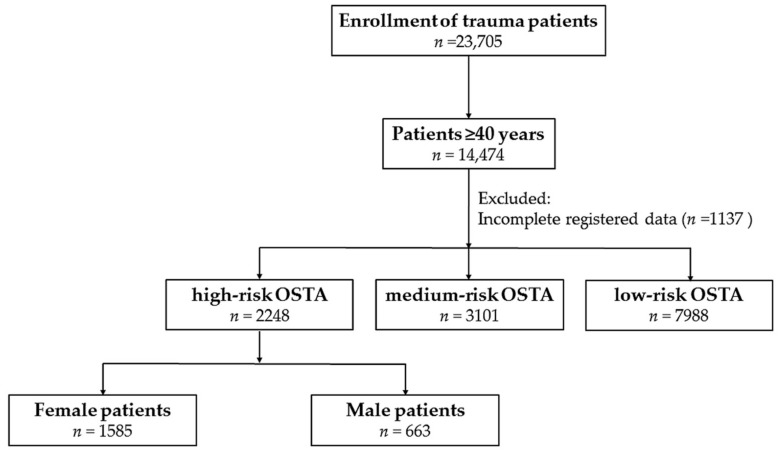
A flow chart presenting the grouping of patients with a high-risk Osteoporosis Self-assessment Tool for Asians (OSTA) score based on their sex.

**Figure 2:**
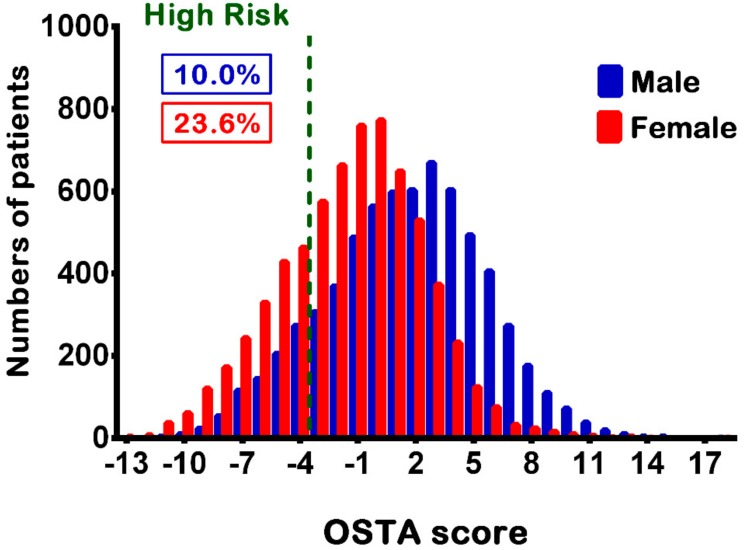
Distribution of the OSTA scores in the female and male patients as well as the percentage of patients having a high-risk OSTA score.

The OSTA score was calculated based on the patients’ age and body weight using the following formula: (body weight (kilogram) − age (year) × 0.2. The study population included patients aged ≥40 years and who had high-risk OSTA scores. Those who had incomplete registered data were excluded from the study (*n* = 1137). Overall, 2248 patients with a high-risk OSTA score including 663 women and 1585 men were included in this study (Figure 1), accounting for 10.0% and 23.6% of the total male and female patients, respectively (Figure 2). The following patient information was retrieved from the trauma registry system: (1) age; (2) body weight (kg) and height (cm); (3) comorbidities such as diabetes mellitus (DM), hypertension (HTN), coronary artery disease, congestive heart failure, and cerebral vascular accident (CVA); (4) blood alcohol concentration (BAC), with a BAC level of 50 mg/dL being arbitrarily defined as the cut-off value for alcohol intoxication; (5) Glasgow coma scale (GCS), which is the summation of scores for eye, verbal, and motor responses with minimum score of 3 indicating deep coma or a brain-dead state and maximum score of 15 indicating a fully awake patient [25], upon arrival to the emergency department; (6) abbreviated injury scale (AIS), which assesses the injury severity on a six-point scale ranging from minor (1-score), moderate (2-score), serious (3-score), severe (4-score), critical (5-score), to un-survivable injury (6-score), and served as an anatomy-based measurement for ranking specific injuries of six predefined body regions in an individual [26]; (7) ISS, which indicates the injury severity of the trauma patient with the summation of the squares of the AIS scores of three most severe injuries [27], expressed as the median and interquartile range [IQR, Q1–Q3]); (8) mortality; (9) length of stay (LOS) in the hospital and in the intensive-care unit (ICU); and (10) information regarding whether the patient had been admitted to the ICU or not. 

### 2.3. Statistical Analysis

We used the IBM SPSS software for Windows, version 20.0 (IBM Corp., Armonk, NY, USA) for statistical analysis. Pearson’s chi-squared, chi-squared, and two-sided Fisher’s exact tests were used to compare the categorical data. The odds ratios (ORs) of the associated conditions and injuries of the patients were calculated with 95% confidence intervals (CIs). The continuous data were expressed as mean ± standard deviation and analyzed using the unpaired Student’s *t*-test and Mann–Whitney *U*-test for normally and non-normally distributed data, respectively. In order to eliminate the confounding effects of the non-random assignment of patients based on their OSTA scores, when assessing the patient outcomes, the NCSS software v.10 (NCSS Statistical Software, Kaysville, UT, USA) was used to calculate the propensity score based on age, comorbidity, mechanism and ISS. A separate 1:1 matched set of comparable study populations for the male vs. female patients was created using the greedy method according to the propensity scores. The greedy method selects randomly a treated subject at first. The untreated subject with closest propensity score to that of this randomly selected treated subject is chosen for matching. A binary logistic regression was used to assess the effect of sex-related groups on patient outcomes. A *p*-value of <0.05 was considered to be statistically significant.

## 3. Results 

### 3.1. Characteristics of Patients

As shown in Table 1, in the studied population, the mean age of female patients is lower than that of the male patients (80.6 ± 6.9 [range 58–102] vs. 82.1 ± 6.3 [range 59–99], *p* < 0.001). As expected, the mean body weight and height were significantly lower in the female patients than that of the male patients. Although female patients demonstrated higher rates of pre-existing DM and HTN than their male counterparts, higher rates of pre-existing CVA were present in the male patients when compared to the female patients. With regard to the mechanism of injury, female patients displayed lower odds of sustaining motorcycle or bicycle accidents, but higher odds of fall-related accidents than their male counterparts. The mean age of motorcyclists and cyclists was significantly lower than those who had a fall among the female patients (Appendix A Table A1). In addition, the mean age of motorcyclists, but not cyclists, was significantly lower than those who had a fall among the male patients. Although both groups had low incidence of positive BAC, the number of female patients was less than that of the male patients. Female patients had a higher GCS than male patients, but the difference in score was less than one point. In the analysis of patients with an AIS of ≥3, which is indicative of a serious injury, female patients had higher odds of extremity injuries than male patients; while the male patients had higher odds of head and neck and thorax injuries than the female patients. Female patients had a significantly lower ISS than the male patients, with most of the female patients demonstrating an ISS of <16, although few female patients had an ISS of 16–24.

### 3.2. Outcome of Propensity-Score Matched Patients

**Figure 3:**
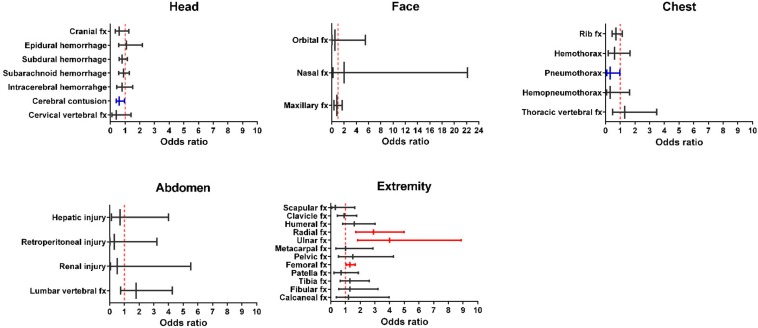
Odds of associated illness between the propensity-score matched female and male patients with high-risk OSTA score. fx = fracture.

A separate propensity score-matched population was created to minimize the selection bias during outcome assessment. Moreover, the study compared the different trauma patterns in various body parts of the male and female patients, including the head, face, thorax, abdomen and extremities. Female patients displayed lower odds of having cerebral contusion (OR, 0.6; 95% CI, 0.40–0.97; *p* = 0.036) and pneumothorax (OR, 0.3; 95% CI, 0.08–0.97; *p* = 0.031) than male patients (Figure 3 and Appendix A Table A2). In contrast, female patients demonstrated higher odds of radial (OR, 2.9; 95% CI, 1.70–4.99; *p* < 0.001), ulnar (OR, 4.0; 95% CI, 1.84–8.87; *p* < 0.001), and femoral (OR, 1.3; 95% CI, 1.05–1.67; *p* = 0.018) fracture than male patients. In this selected patient cohort, 573 well-balanced pairs of female and male patients demonstrated no significant differences in regard to their age, comorbidity, mechanism and ISS (Table 2). Comparative assessment of the clinical outcomes of these 573 well-balanced pairs of patients with high-risk OSTA score revealed that female patients (OR, 0.5; 95% CI, 0.29–0.90; *p* = 0.019) demonstrated lower mortality rates than male patients. However, the comparison of propensity-score matched female patients vs. male patients revealed that there were no significant difference in regard to the LOS in the hospital, rates of ICU admission, and LOS in the ICU (Table 3).

## 4. Discussion

This study compared the impact of sex dimorphism on the clinical outcomes of patients with high-risk OSTA scores. It showed that male patients were significantly older, had higher incidence of comorbidities and head/neck and thoracic injuries, and were more frequently injured in motorcycle accidents when compared to their female counterparts. In contrast, female patients had higher incidences of extremity injuries. Despite adjusting for confounding factors, including age, pre-existing comorbidities, trauma mechanism, and injury severity, female patients still demonstrated a 0.5-fold lower odds of mortality than male patients. 

These results indicated that sexual dimorphism had an impact on trauma patterns and the clinical outcomes of patients. There were various associated injuries in female and male patients. In this study, the male patients had higher odds of cerebral contusion and pneumothorax, whereas the female patients had higher odds of radial, ulnar and femoral fractures. The different proportion of trauma injuries may be related to the higher rate of motorcycle accidents in male patients and a higher rate of fall-related accidents in female patients. Notably, those with motorcycle accidents were younger than those falling when walking, and the cyclists were significantly younger than those who had a fall in the female, but not male, patients. Furthermore, another explanation for the different associated injuries may be attributed to the fact that mean body weight and height were significantly lower in the female patients than in male patients. 

Furthermore, head and thoracic injuries were reported to be the independent risk factors for mortality among the 10,607 motorcycle riders [28]. Alexander et al. reported that elderly patients with multiple rib fractures and cardiopulmonary disease had a significant risk of mortality [29]. Although the ISS was adjusted in the selected propensity-score matched populations, the risks of mortality in patients with an AIS value categorized as serious to critical injury in different injured body regions may not be the same, even after controlling for the potential confounding factors [30]. Moreover, the different odds of associated illness of female and male patients prone to extremity fractures and cerebral contusion as well as pneumothorax, respectively, did not fully explain the discrepancy of the mortality number between sexes.

Moreover, hormonal differences may result in survival advantage. Estrogen improves myocardial and hepatocellular functions and decreases lung congestion after trauma. In contrast, endogenous testosterone depresses the immune response and causes the impairment of organ functions following trauma and blood loss [31]. Some studies using human and animal models have revealed that the sex hormones play an important role in the body’s response and affect clinical outcomes [31,32,33]. The Women’s Health Initiative has validated the value of long-term hormone therapy in women at risk of osteoporosis, in which the female patients in the studied population had benefited from this policy and had a survival advantage [34]. However, further prospective studies are needed to confirm the protective role of hormones in mortality based on the observed sex differences of patients with high-risk OSTA scores.

This study had several limitations. First, its retrospective design resulted in an inherent selection bias, even while adopting a method of propensity-score matching. Second, the descriptive study had no data regarding the indications for admission into or discharge from the ward and ICU, which may have resulted in a selection bias. Third, patients with trauma who died outside the hospital, those who were discharged against the advice of medical personnel from the emergency department, and those who were not admitted in the emergency department were not included in this study, which may have also caused a selection bias. Fourth, the lack of important data regarding physical activity, nutrition status, and cognitive function in the trauma registry system may result in bias in the analysis of outcomes. Furthermore, a low frequency of some associated illnesses may lead to bias in the assessment of the odds of relative risk. Also, the impact of “care manager” nurses, who may attribute a specialized role into the primary health care system, on the outcome was not assessed in this study [35]. Finally, these results were obtained from a study population at a single level I trauma center in southern Taiwan, and hence may not be generalized to other populations.

## 5. Conclusions

In this study, female patients with a high-risk OSTA score showed different injury patterns and presented lower mortality rates than their male counterparts, even after controlling for potential confounding factors.

## Figures and Tables

**Table 1:** Demographics and injury characteristics of male and female patients with high-risk OSTA scores.

Variables	Female, *n* = 1585	Male, *n* = 663	Odds Ratio (95% CI)	*p*
Age [range] (years)	80.6 ± 6.9 [58–102]	82.1 ± 6.3 [59–99]	-	<0.001
Body weight (kg)	48.6 ± 7.1	52.7 ± 6.7	-	<0.001
Body height (cm)	151.7 ± 5.7	162.9 ± 6.1	-	<0.001
Co-morbidity, *n* (%)				
DM	400 (25.2)	92 (13.9)	2.1 (1.64–2.68)	<0.001
HTN	962 (60.7)	310 (46.8)	1.8 (1.47–2.11)	<0.001
CAD	156 (9.8)	59 (8.9)	1.1 (0.82–1.53)	0.488
CHF	44 (2.8)	17 (2.6)	1.1 (0.62–1.91)	0.778
CVA	151 (9.5)	91 (13.7)	0.7 (0.50-0.87)	0.003
Mechanism, *n* (%)				
Motor vehicle	9 (0.6)	3 (0.5)	1.3 (0.34–4.66)	1.000
Motorcycle	133 (8.4)	132 (19.9)	0.4 (0.28–0.48)	<0.001
Bicycle	74 (4.7)	53 (8.0)	0.6 (0.39–0.81)	0.002
Pedestrian	56 (3.5)	18 (2.7)	1.3 (0.77–2.25)	0.321
Fall	1277 (80.6)	434 (65.5)	2.2 (1.79–2.68)	<0.001
Penetrating injury	7 (0.4)	6 (0.9)	0.5 (0.16–1.45)	0.222
Struck by/against	29 (1.8)	17 (2.6)	0.7 (0.39–1.30)	0.262
BAC ≥ 50 mg/dL, *n* (%)	1 (0.1)	5 (0.8)	0.1 (0.01–0.71)	0.010
GCS	14.4 ± 1.9	14.1 ± 2.2	-	0.006
≤8	49 (3.1)	32 (4.8)	0.6 (0.40–0.99)	0.044
9–12	63 (4.0)	40 (6.0)	0.6 (0.43–0.97)	0.033
≥13	1473 (92.9)	591 (89.1)	1.6 (1.17–2.19)	0.003
AIS ≥ 3, *n* (%)				
Head/Neck	270 (17.0)	188 (28.4)	0.5 (0.42–0.64)	<0.001
Face	0 (0.0)	0 (0.0)	-	-
Thorax	34 (2.1)	40 (6.0)	0.3 (0.21–0.54)	<0.001
Abdomen	15 (0.9)	5 (0.8)	1.3 (0.46–3.47)	0.658
Extremity	991 (62.5)	327 (49.3)	1.7 (1.43–2.06)	<0.001
ISS, median (IQR)	9 (9–9)	9 (9–13)	-	0.001
<16	1369 (86.4)	512 (77.2)	1.9 (1.48–2.36)	<0.001
16–24	161 (10.2)	118 (17.8)	0.5 (0.40–0.68)	<0.001
≥25	55 (3.5)	33 (5.0)	0.7 (0.44–1.07)	0.093
Mortality, *n* (%)	44 (2.8)	40 (6.0)	0.4 (0.29–0.69)	<0.001
LOS in hospital (days)	9.6 ± 8.3	11.2 ± 11.4	-	0.001
ICU admission, *n* (%)	308 (19.4)	197 (29.7)	0.6 (0.46–0.70)	<0.001
LOS in ICU (days)	7.1 ± 9.7	8.6 ± 10.0	-	0.097

AIS = abbreviated injury scale; BAC = blood alcohol concentration; CAD = coronary artery disease; CI = confidence interval; CVA = cerebral vascular accident; DM = diabetes mellitus; GCS = Glasgow coma scale; HTN = hypertension; ICU = intensive care unit; IQR = interquartile range; ISS = injury severity score; LOS = length of stay; OR = odds ratio.

**Table 2:** Covariates of male and female patients with high-risk OSTA scores before and after propensity-score matching analyses (1:1 matching via greedy method).

Variables	Before Matching				After Matching			
	Female, *n* = 1585	Male, *n* = 663	Odds Ratio (95% CI)	*p*	Female, *n* = 573	Male, *n* = 573	Odds Ratio (95% CI)	*p*
Age (years)	80.6 ± 6.9	82.1 ± 6.3	-	<0.001	81.4 ± 6.3	81.8 ± 6.4	-	0.402
Co-morbidity, *n* (%)								
DM	400 (25.2)	92 (13.9)	2.1 (1.64–2.68)	<0.001	80 (1.4)	80 (1.4)	1.0 (0.72–1.40)	1.000
HTN	962 (60.7)	310 (46.8)	1.8 (1.47–2.11)	<0.001	282 (49.2)	282 (49.2)	1.0 (0.79–1.26)	1.000
CAD	156 (9.8)	59 (8.9)	1.1 (0.82–1.53)	0.488	49 (8.6)	49 (8.6)	1.0 (0.66–1.51)	1.000
CHF	44 (2.8)	17 (2.6)	1.1 (0.62–1.91)	0.778	12 (2.1)	12 (2.1)	1.0 (0.45–2.25)	1.000
CVA	151 (9.5)	91 (13.7)	0.7 (0.50–0.87)	0.003	73 (12.7)	73 (12.7)	1.0 (0.71–1.42)	1.000
Mechanism, *n* (%)								
Motor vehicle	9 (0.6)	3 (0.5)	1.3 (0.34–4.66)	1.000	1 (0.2)	1 (0.2)	1.0 (0.06–16.03)	1.000
Motorcycle	133 (8.4)	132 (19.9)	0.4 (0.28–0.48)	<0.001	80 (14.0)	80 (14.0)	1.0 (0.72–1.40)	1.000
Bicycle	74 (4.7)	53 (8.0)	0.6 (0.39–0.81)	0.002	39 (6.8)	39 (6.8)	1.0 (0.63–1.58)	1.000
Pedestrian	56 (3.5)	18 (2.7)	1.3 (0.77–2.25)	0.321	15 (2.6)	15 (2.6)	1.0 (0.48–2.07)	1.000
Fall	1277 (80.6)	434 (65.5)	2.2 (1.79–2.68)	<0.001	427 (74.5)	427 (74.5)	1.0 (0.77–1.30)	1.000
Penetrating injury	7 (0.4)	6 (0.9)	0.5 (0.16–1.45)	0.222	1 (0.2)	1 (0.2)	1.0 (0.06–16.03)	1.000
Struck by/against	29 (1.8)	17 (2.6)	0.7 (0.39–1.30)	0.262	10 (1.7)	10 (1.7)	1.0 (0.41–2.42)	1.000
ISS, median (IQR)	9 (9–9)	9 (9–13)	-	0.001	9 (9–13)	9 (9–13)	-	0.400

CAD = coronary artery disease; CI = confidence interval; CVA = cerebral vascular accident; DM = diabetes mellitus; HTN = hypertension; IQR = interquartile range; ISS = injury severity score; OR = odds ratio.

**Table 3:** Clinical outcomes of male and female patients with high-risk OSTA scores assessed using the propensity-score matching analyses after adjusting for age, comorbidity, mechanism, and injury severity score (ISS).

Variables	Propensity-Score Matched Cohort			
	Female, *n* = 573	Male, *n* = 573	Odds Ratio (95% CI)	*p*
Mortality, *n* (%)	19 (3.3)	36 (6.3)	0.5 (0.29–0.90)	0.019
LOS in hospital (days)	10.4 ± 9.3	11.3 ± 11.5	-	0.154
ICU admission, *n* (%)	143 (25.0)	172 (30.0)	0.8 (0.60–1.01)	0.055
LOS in ICU (days)	7.6 ± 10.0	8.8 ± 10.4	-	0.286

## Abbreviations

The following abbreviations are used in this manuscript:

AIS	abbreviated injury scale
BMI	body mass index
CAD	coronary artery disease
CHF	congestive heart failure
CIs	confidence intervals
CVA	cerebrovascular accident
DM	diabetes mellitus
ED	emergency department
GCS	Glasgow coma scale
HTN	hypertension
ICU	intensive-care unit
IQR	interquartile range
ISS	injury-severity score
LOS	length of stay
ORs	odds ratios
OSTA	Osteoporosis Self-Assessment Tool for Asians

## Appendix A

**Table A1:** Trauma patterns in different body parts of male and female patients with high-risk OSTA scores.

Variables	Female, *n* = 573	Male, *n* = 573	Odds Ratio (95% CI)	*p*
Head trauma, *n* (%)				
Cranial fracture	15 (2.6)	23 (4.0)	0.6 (0.33–1.25)	0.187
Epidural hematoma (EDH)	19 (3.3)	17 (3.0)	1.1 (0.58–2.18)	0.735
Subdural hematoma (SDH)	87 (15.2)	101 (17.6)	0.8 (0.61–1.15)	0.264
Subarachnoid hemorrhage (SAH)	49 (8.6)	56 (9.8)	0.9 (0.58–1.29)	0.474
Intracerebral hematoma (ICH)	20 (3.5)	24 (4.2)	0.8 (0.45–1.52)	0.539
Cerebral contusion	35 (6.1)	54 (9.4)	0.6 (0.40–0.97)	0.036
Cervical vertebral fracture	3 (0.5)	8 (1.4)	0.4 (0.10–1.41)	0.130
Maxillofacial trauma, *n* (%)				
Orbital fracture	1 (0.2)	2 (0.3)	0.5 (0.05–5.52)	1.000
Nasal fracture	2 (0.3)	1 (0.2)	2.0 (0.18–22.16)	1.000
Maxillary fracture	12 (2.1)	15 (2.6)	0.8 (0.37–1.72)	0.559
Mandibular fracture	2 (0.3)	0 (0.0)	-	0.500
Thoracic trauma, *n* (%)				
Rib fracture	33 (5.8)	45 (7.9)	0.7 (0.45–1.14)	0.159
Hemothorax	5 (0.9)	9 (1.6)	0.6 (0.18–1.66)	0.282
Pneumothorax	3 (0.5)	11 (1.9)	0.3 (0.08–0.97)	0.031
Hemopneumothorax	2 (0.3)	6 (1.0)	0.3 (0.07–1.65)	0.287
Thoracic vertebral fracture	9 (1.6)	7 (1.2)	1.3 (0.48–3.49)	0.615
Abdominal trauma, *n* (%)				
Hepatic injury	2 (0.3)	3 (0.5)	0.7 (0.11–4.00)	1.000
Splenic injury	4 (0.7)	0 (0.0)	-	0.124
Retroperitoneal injury	1 (0.2)	3 (0.5)	0.3 (0.03–3.20)	0.624
Renal injury	1 (0.2)	2 (0.3)	0.5 (0.05–5.52)	1.000
Lumbar vertebral fracture	14 (2.4)	8 (1.4)	1.8 (0.74–4.25)	0.196
Extremity trauma, *n* (%)				
Scapular fracture	2 (0.3)	6 (1.0)	0.3 (0.07–1.65)	0.287
Clavicle fracture	16 (2.8)	18 (3.1)	0.9 (0.45–1.76)	0.728
Humeral fracture	23 (4.0)	15 (2.6)	1.6 (0.80–3.01)	0.187
Radial fracture	52 (9.1)	19 (3.3)	2.9 (1.70–4.99)	<0.001
Ulnar fracture	31 (5.4)	8 (1.4)	4.0 (1.84–8.87)	<0.001
Metacarpal fracture	7 (1.2)	7 (1.2)	1.0 (0.35–2.87)	1.000
Pelvic fracture	9 (1.6)	6 (1.0)	1.5 (0.53–4.26)	0.436
Femoral fracture	291 (50.8)	251 (43.8)	1.3 (1.05–1.67)	0.018
Patella fracture	6 (1.0)	9 (1.6)	0.7 (0.23–1.88)	0.436
Tibia fracture	18 (3.1)	14 (2.4)	1.3 (0.64–2.63)	0.473
Fibular fracture	12 (2.1)	9 (1.6)	1.3 (0.56–3.21)	0.509
Calcaneal fracture	6 (1.0)	5 (0.9)	1.2 (0.37–3.96)	0.762

**Table A2:** Comparison of the mean age of motorcyclists and cyclists against incidence of those who had a fall among the female and male patients.

Male	Motorcycle, *n* = 132	Bicycle, *n* = 53	Fall, *n* = 434	Motorcycle vs. Fall (*p*)	Bicycle vs. Fall (*p*)
Age [range] (years)	80.6 ± 5.7 [68–94]	82.4 ± 5.6 [68–98]	82.7 ± 6.5 [59–99]	0.001	0.770
